# Combination of polygonatum sibiricum and hedyotis diffusa induces pyroptosis in non-small cell lung cancer cells via the TLR4/NLRP3/Caspase-1/GSDMD pathway: an *in vitro* and *in vivo* study using medicated serum and herbal extract

**DOI:** 10.3389/fphar.2026.1793565

**Published:** 2026-07-06

**Authors:** Lei Mao, Lingying Jiang, Ye Zhou, Shouhang Xu, Tingting Lou, Weiyong Lu, Gaoyuan Zhu, Jianwu Chen

**Affiliations:** 1 Jiangshan Traditional Chinese Medicine Hospital, Quzhou, China; 2 Tongde Hospital of Zhejiang Province, Hangzhou, China; 3 Pu Jiang Second Hospital, Jinhua, China

**Keywords:** cell, Hedyotis diffusa, non-small cell lung cancer, Polygonatum, pyroptosis

## Abstract

**Objective:**

To study the effects of Polygonatum-Hedyotis diffusa on non-small cell lung cancer and the mechanism of action.

**Methods:**

The CCK-8 assay was used to investigate the effects of different ratios of Polygonatum-Hedyotis diffusa containing serum on the proliferation of A549 cells, and the optimal ratios were screened out for subsequent experiments. The scratch and invasion assays were used to evaluate the inhibition of migration and invasion of A549 cells by Polygonatum-Hedyotis diffusa containing serum. Flow cytometry was used to analyze the effect of Polygonatum-Hedyotis diffusa containing serum on the apoptosis of A549 cells, and Western blot assay was used to analyze the protein expression levels of TLR4, NLRP3, Caspase-1, GSDMD, IL-1β, and IL-18. Finally, mice were injected subcutaneously with A549 cells *in vivo* to form solid tumors to further validate the *in vivo* inhibitory effect of Polygonatum-Hedyotis diffusa on non-small-cell lung cancer, and Western blot experiments were used to re-validate the levels of TLR4, NLRP3, Caspase-1, GSDMD, IL-1β, and IL-18 protein expression in tumor cells.

**Result:**

CCK-8 experiments were used to demonstrate that the 3:1 ratio of Polygonatum-Hedyotis diffusa containing serum had the best inhibitory effect on A549 cells. Scratch and invasion experiments verified that the 3:1 ratio of Polygonatum-Hedyotis diffusa containing serum inhibited the migration and invasion ability of A549. Flow cytometry results illustrated that 3:1 ratio of Polygonatum-Hedyotis diffusa containing serum induced apoptosis in A549 cells, and Western blot experiments verified that the expression levels of TLR4, NLRP3, Caspase-1, GSDMD, IL-1β, and IL-18 proteins were up-regulated. *In vivo* animal experiments further validated Polygonatum-Hedyotis diffusa for the treatment of non-small cell lung cancer, and Western blot verified the upregulation of the protein expression levels of TLR4, NLRP3, Caspase-1, GSDMD, IL-1β, and IL-18 in tumor cells.

**Conclusion:**

3:1 ratio of Polygonatum-Hedyotis diffusa containing serum can inhibit the proliferation of A549 cells through cellular pyroptosis, providing a new idea for the treatment of non-small cell lung cancer. The upregulation of these proteins suggests the involvement of the pathway, but causal evidence is not provided in this study.

## 1 Introduction

Lung cancer is one of the malignant tumors with high morbidity and mortality in China, with non-small cell lung cancer (NSCLC) accounting for about 85% of all lung cancer cases (Li et al., 2024; Peng et al., 2024; Bade and Dela Cruz, 2020). Non-small cell lung cancer can be further classified into squamous cell carcinoma, adenocarcinoma, and undifferentiated large cell carcinoma (Li et al., 2024; Peng et al., 2024; Bade and Dela Cruz, 2020). Current treatments for NSCLC include chemotherapy, surgical resection, and immunotherapy. Although these approaches can delay disease progression, issues such as postoperative recurrence, metastasis, and drug resistance remain significant challenges (Lai et al., 2024; Zhong and Zhu, 2024; Zhang, 2024). Therefore, exploring more effective drugs for the treatment of non-small cell lung cancer remains crucial. Lung adenocarcinoma is the most common pathological subtype of NSCLC. In this study, human lung adenocarcinoma A549 cells were selected as the research model. As a classic and widely used cell line in NSCLC research, A549 cells exhibit stable biological characteristics, and the findings of this study have direct reference value for the clinical diagnosis and treatment of lung adenocarcinoma.

**FIGURE 1:**
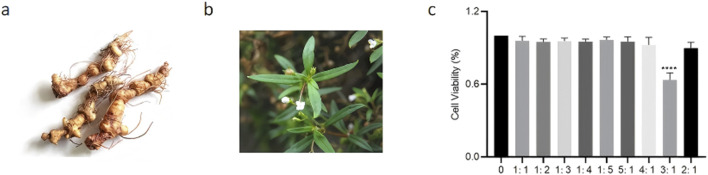
Effects of different ratios of drug on the proliferation of A549 cells. **(a)** Representative images of Polygonatum sibiricum. **(b)** Representative images of Hedyotis diffusa. **(c)** CCK-8 assay of A549 cells treated with different ratios of medicated serum (10% v/v, 72 h). Data are presented as mean ± SD (n = 6 per group). Statistical analysis: one-way ANOVA with Tukey’s post-hoc test for multiple comparisons. Compared with the control group, ***P < 0.0001. Note: a: Representative images of the Chinese herbal medicine Polygonatum sibiricum; b: Representative images of the Chinese herbal medicine Hedyotis diffusa; c: Compared with the control group, the herbal extract group with a 3:1 ratio of Polygonatum sibiricum to Hedyotis diffusa, ****P < 0.0001.

Polygonatum sibiricum belongs to the Liliaceae family, and its medicinal part is the dried rhizome (Figure 1a). Liu Haitao et al. found that the combination of Polygonatum sibiricum and Scutellaria baicalensis could treat A549 cells by downregulating PON3-induced mitochondrial damage and endoplasmic reticulum stress (Liu et al., 2024). Wang Zongcan et al. also demonstrated that a compound of Polygonatum sibiricum and Astragalus membranaceus could inhibit lung cancer progression by downregulating the apelin-PGC1α-UCP1 signaling pathway. In addition, Polygonatum sibiricum has been shown to inhibit various cancers, including bladder cancer and prostate cancer (Wang et al., 2024; Lv et al., 2024; Yang et al., 2024; Yu et al., 2020).

Hedyotis diffusa belongs to the Rubiaceae family, and its medicinal part is the whole plant (Figure 1b). Existing research has shown that compounds contained in Hedyotis diffusa, such as terpenoids and flavonoids, can exert anti-small cell lung cancer effects, characterized by multiple components, multiple targets, and multiple pathways (Peng et al., 2024; Pang and Zheng, 2021; Chen et al., 2024). Although Polygonatum sibiricum and Hedyotis diffusa have been separately applied in drugs against non-small cell lung cancer, the therapeutic efficacy of their combination for treating non-small cell lung cancer has not been reported.Although the compatibility of Polygonatum sibiricum and Hedyotis diffusa is not directly recorded in classic traditional Chinese medicine prescriptions, it conforms to the core principle of “strengthening healthy Qi and eliminating pathogenic factors in the treatment of non-small cell lung cancer. Polygonatum sibiricum strengthens healthy Qi and benefits Qi to consolidate the root, while Hedyotis diffusa clears heat, detoxifies and dissipates masses to eliminate pathogenic factors. The combination of the two herbs treats both the manifestation and root cause, providing a new strategy for traditional Chinese medicine compatibility in clinical lung cancer treatment. At present, the effect and mechanism of their combination in regulating cell pyroptosis have not been reported. Therefore, relevant research was carried out in this study.

## 2 Materials and instruments

### 2.1 Experimental drug

Both Polygonatum sibiricum and Hedyotis diffusa were purchased from Quzhou Nankong Traditional Chinese Medicine Co., Ltd. The reflux extraction method was used to prepare herbal solutions of Polygonatum sibiricum and Hedyotis diffusa at the ratios of 1:1, 1:2, 1:3, 1:4, 1:5, 5:1, 4:1, 3:1, and 2:1. The prepared solutions were administered to SD rats by gavage at a dose of 16 g/kg/d. A control group received an equal volume of physiological saline. The rats were divided into groups corresponding to the herbal solution ratios (1:1, 1:2, 1:3, 1:4, 1:5, 5:1, 4:1, 3:1, 2:1) and a control group. Gavage was performed twice daily at 12-h intervals for 14 consecutive days. Blood was collected 1 hour after the last administration, and herbal extract was obtained from all herbal solution groups and the control group. Blood samples were collected from rats in all groups 1 h after the last administration. herbal extract was isolated and prepared by routine methods in each gavage group administered with different ratios of Polygonatum sibiricum–Hedyotis diffusa herbal solution. Blank rat serum was obtained from the normal saline gavage control group using the same blood separation and preparation procedure. All sera were sterilized by filtration through a 0.22 μm sterile filter membrane and stored at −80 °C until use, providing control and treatment samples for subsequent cell experiments.

### 2.2 Cells and experimental animals

The human non-small cell lung cancer cell line A549 was purchased from the Shanghai Cell Bank of the Chinese Academy of Sciences. Specific pathogen-free (SPF) BALB/c-nude male mice (6–8 weeks old, weighing 18–22 g) and SD rats (weighing 230–250 g) were purchased from Shanghai Bikai Keyi Biotechnology Co., Ltd. All animals were housed in the barrier laboratory of the Animal Experiment Center at Zhejiang Chinese Medical University (Facility Use License No.: SYXK (Zhe) 2021-0012). All animal experimental procedures were conducted in accordance with the guidelines of the Experimental Research Institute of Zhejiang Chinese Medical University (Ethics Approval No.: IACUC-202406-20). (Although the use of rat-derived medicated serum on human A549 cells may introduce potential cross-species effects, this medicated serum approach is a well-established technique in *in vitro* pharmacology studies of traditional Chinese medicine, as it captures the active metabolite complex after oral administration. Importantly, our subsequent *in vivo* experiment (nude mice orally administered the same herbal extract) verified the key anti-tumor effects and molecular changes in a same-species system, and the results were consistent with the *in vitro* findings, which partially mitigates concerns about cross-species interference. Readers should consider this methodological limitation when interpreting the *in vitro* data).

### 2.3 Reagents

The CCK-8 reagent was purchased from Dojindo Laboratories, Japan (Batch No.: 11875176). The cell apoptosis kit was purchased from Sigma (Batch No.: APOAF). The BCA protein concentration assay kit (Product No.: P0010) was purchased from Beyotime Biotechnology. NLRP3 (Batch No.: ab263899), IL-1β (Batch No.: ab254360), IL-18 (Batch No.: ab207323), and β-actin (Batch No.: ab8266) were all purchased from Abcam. TLR4 (Batch No.: 48-2300) was purchased from Thermo Fisher. Caspase-1 (Batch No.: 4199) and GSDMD (Batch No.: 36425) were purchased from Cell Signaling Technology. The Ki67 antibody was purchased from Cell Signaling Technology.

### 2.4 Instrument

SpectraMax M5/M5e multi-mode microplate reader (Model: Molecular Devices); inverted optical microscope (Model: CX43, Brand: Olympus, Japan); flow cytometer (Model: BD Accuri C6 Plus, Brand: BD, United States); protein electrophoresis and transfer system (Model: 1658033, Brand: Bio-Rad, United States).

## 3 Methods

### 3.1 CCK-8 experiment

A549 cells in the logarithmic growth phase were seeded into 96-well plates at a density of 1 × 10^4^ cells per well and cultured for 24 h. Subsequently, the cells were treated with 10% herbal extract from the Polygonatum sibiricum to Hedyotis diffusa groups at ratios of 1:1, 1:2, 1:3, 1:4, 1:5, 5:1, 4:1, 3:1, and 2:1, The control group was given an equal volume of 10% blank rat serum. All experimental groups and the control group were cultured for another 72 h, and all other operating conditions remained identical. After removing the original culture medium, 100 µL of basic medium containing 10% CCK-8 reagent was added to each well. The plates were incubated at 37 °C for 1 h, and the absorbance at 450 nm was measured using a microplate reader to assess the effect of the drug treatments on the proliferation of A549 cells.

### 3.2 Cell scratch test

A549 cells were digested with trypsin, and the cell pellet was resuspended in culture medium. Then, 2 × 10^5^ cells were seeded into a 6-well plate. When the cell density reached approximately 95%, a scratch was made using a 100 µL pipette tip and photographed. Subsequently, 10% herbal extract of Polygonatum sibiricum to Hedyotis diffusa at a 3:1 ratio was added, and the cells were further cultured for 72 h. The same location was photographed again, and the migration distance was finally calculated using ImageJ.

### 3.3 Cell invasion experiment

After drug treatment, cells were seeded onto Matrigel containing invasion upper chamber, and 600 ul of cell culture medium (containing 10% FBS) was added to the lower chamber for 72 h. Residual cells in the upper chamber were wiped off, fixed with paraformaldehyde for 20–30 min, stained with crystal violet for 20–30 min, washed with water, inverted microscope, and counted using ImageJ.

### 3.4 Cell apoptosis experiment

Take A549 cells in logarithmic growth stage and digest them with trypsin. Resuspend the cell pellet in the culture medium. Inoculate 9 × 10^4^ cells into a six well plate and culture for 24 h. Add 10% 3:1 ratio of Huangjing White Hedyotis diffusa drug containing serum and continue to incubate for 72 h. Collect the cells. Incubate the cells with 5 μ L Annexin-V-FITC and 10 μ L PI buffer in the dark at room temperature for 15 min, flow cytometry, and analyze the data using FlowJo software.

### 3.5 Protein immunoblotting experiment

Take A549 cells in logarithmic growth phase for trypsin digestion, resuspend the cell pellet in culture medium, and inoculate 9 × 10^4^ cells into a six well plate. Cultivate for 24 h, add 10% 3:1 ratio of Huangjing: Hedyotis diffusa drug containing serum, continue to incubate for 72 h, and collect the cell pellet. RIPA lysis buffer was added to the cell pellet to lyse the cells, and protein quantification was performed using BCA assay kit. Prepare 10% gel - sample loading (add 5 × SDS-PAGE for cell protein precipitation, 10 ul per hole) - gel running (80V upper layer gel running, 120V lower layer gel running) - sealing (sealed with milk for 1 hour) - primary antibody incubation (primary antibody incubated at 4 °C overnight) - secondary antibody incubation - development (exposed and collected by chemiluminescence instrument), and use ImageJ to analyze the gray value, Prism to analyze the difference.

### 3.6 Animal experimentation

Six-to eight-week-old BALB/c-nude male mice were subcutaneously inoculated with A549 cells. When the tumor volume reached approximately 100 mm^3^, oral gavage administration was initiated at a dose of 16 g/kg, administered once every 2 days. The body weight and tumor volume of the mice were recorded simultaneously. On day 14 after the start of administration, the mice were euthanized. Tumors were collected and weighed. Major organs were subjected to HE staining to evaluate the toxicity of the drug in mice. Another portion of the tumor tissue was taken for protein extraction to perform Western blot (WB) analysis.

### 3.7 Statistical methods

All experimental data were statistically analyzed using Prism and ImageJ software. Each experiment was independently repeated three times, and the data are presented as Mean ± SD. Comparisons between two groups were performed using the t-test. A P-value <0.05 was considered statistically significant.

## 4 Results

### 4.1 The herbal extract of Polygonatum sibiricum and Hedyotis diffusa at a 3:1 ratio inhibits the growth of A549 cells

Polygonatum sibiricum and Hedyotis diffusa at the specified ratios (1:1, 1:2, 1:3, 1:4, 1:5, 5:1, 4:1, 3:1, and 2:1) were weighed and separately placed in 70% ethanol. After reflux extraction and filtration, the filtrates were collected. The filtrates were administered to SD rats by continuous gavage for 14 days to obtain Polygonatum sibiricum-Hedyotis diffusa herbal extract. The effects of the herbal extract at different ratios on the proliferation of A549 cells were evaluated using the CCK-8 assay. The CCK-8 results indicated that the herbal extract at a 3:1 ratio of Polygonatum sibiricum to Hedyotis diffusa inhibited the growth of A549 cells (as shown in Figure 1c).

To quantitatively evaluate the potency of the 3:1 medicated serum, we analyzed its inhibitory effect on A549 cell proliferation across a range of concentrations (2.5%, 5%, 10%, 15%, 20%, and 30% v/v) based on CCK-8 assay data. The IC_50_ value at 72 h was calculated using nonlinear regression analysis, which was approximately 12.3% (v/v) (95% confidence interval: 10.8%–13.9%). This value provides a reference for comparing the anti-proliferative efficacy of the Polygonatum-Hedyotis diffusa combination with other agents in future studies.

### 4.2 The herbal extract of polygonatum sibiricum and hedyotis diffusa at a 3:1 ratio inhibits the migratory and invasive abilities of A549 cells

**FIGURE 2:**
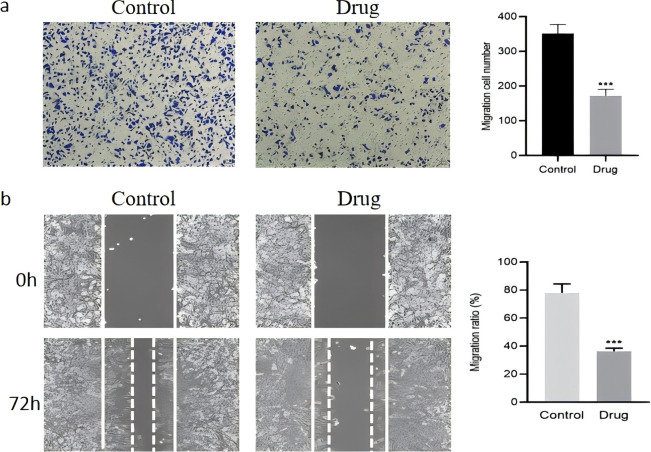
Effect of the 3:1 ratio medicated serum on A549 cell invasion **(a)** and migration **(b)**. Data are presented as mean ± SD (n = 3 independent experiments). Statistical analysis: independent samples t-test (normality and homogeneity of variance verified). ***P < 0.001 compared with the control group. Note: a: Compared with the Control group, the Drug group, ***P < 0.001; b: Compared with the control group, the Drug group, ***P < 0.001.

The CCK-8 assay demonstrated that the herbal extract of Polygonatum sibiricum and Hedyotis diffusa at a 3:1 ratio could inhibit the proliferation of A549 cells. Therefore, we selected the 3:1 ratio herbal extract for Transwell and wound healing assays on A549 cells. The Transwell assay was used to evaluate the effect of the 3:1 herbal extract on the invasive ability of A549 cells. Compared with the number of invasive cells in the control group (351 ± 27), the cell count (171 ± 19) after 72 h of treatment significantly decreased (*P* < 0.001), indicating that the 3:1 herbal extract inhibits the invasive ability of A549 cells. Additionally, a wound healing assay was performed. The results showed that the treatment successfully inhibited the migration of A549 cells, reducing the cell migration rate from 77.98% ± 6.42%–36.26% ± 2.31% (*P* < 0.001) (as shown in Figure 2). In summary, the herbal extract of Polygonatum sibiricum and Hedyotis diffusa at a 3:1 ratio can inhibit the migratory and invasive abilities of A549 cells.

### 4.3 Effect of Polygonatum sibiricum-Hedyotis diffusa medicated serum (3:1) on A549 cell death detected by Annexin V/PI staining

**FIGURE 3:**
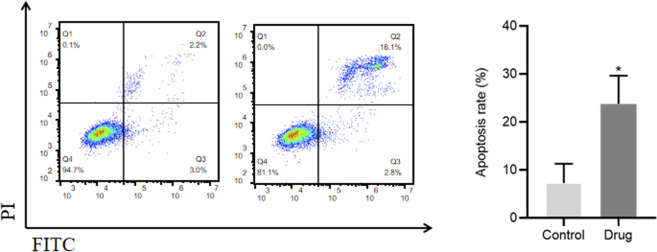
Annexin V/PI staining detecting cell death in A549 cells after treatment with the 3:1 medicated serum for 72 h. Data are presented as mean ± SD (n = 3 independent experiments). Statistical analysis: independent samples t-test. *P < 0.05 compared with the control group. Note: Annexin V/PI cannot distinguish between apoptosis and pyroptosis; therefore, the term “cell deathˮ is used.

Flow cytometry assay was performed to evaluate the apoptotic effect of Polygonatum sibiricum-Hedyotis diffusa herbal extract at a ratio of 3:1 on A549 cells after 72 h of treatment. The results, as shown in Figure 3, showed that the apoptotic rate in the treatment group was significantly higher than that in the control group (apoptotic rate: 7.23% ± 4.05% in the control group vs. 23.77% ± 5.88% in the drug treatment group, P < 0.05). These findings suggest that Polygonatum sibiricum-Hedyotis diffusa herbal extract at a ratio of 3:1 can induce apoptosis of A549 cells.

### 4.4 Detection of the effect of Polygonatum sibiricum-Hedyotis diffusa herbal extract at a ratio of 3:1 on the expression of pyroptosis-Related Proteins by Western blot assay

**FIGURE 4:**
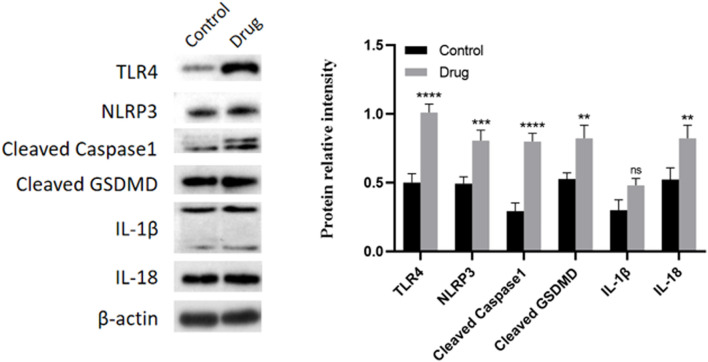
Western blot analysis of TLR4, NLRP3, Caspase-1, GSDMD, IL-1β, and IL-18 protein expression in A549 cells after treatment with the 3:1 medicated serum for 72 h. Data are presented as mean ± SD (n = 3 independent experiments). Statistical analysis: independent samples t-test for each protein. **P < 0.01, ***P < 0.001, ****P < 0.0001 compared with the control group.

To further investigate the mechanism underlying the inhibitory effect of Polygonatum sibiricum-Hedyotis diffusa herbal extract (3:1) on the proliferation of A549 cells, Western blot (WB) assay was employed to detect the expression levels of TLR4, NLRP3, Caspase-1, GSDMD, IL-1β and IL-18 proteins in A549 cells after 72 h of treatment with the herbal extract. As shown in Figure 4, compared with the control group, the expression levels of TLR4, NLRP3, Caspase-1, GSDMD, IL-1β and IL-18 in A549 cells were significantly upregulated after drug treatment, with statistically significant differences (P < 0.01, P < 0.001, P < 0.0001). The increased levels of mature IL-1β (approximately 17 kDa) and mature IL-18 suggest inflammasome activation and caspase-1 cleavage, indicating that pyroptosis-related molecular events may be activated.

### 4.5 Inhibitory effect of Polygonatum sibiricum-Hedyotis diffusa herbal extract at a ratio of 3:1 on the growth of A549 Cell-Derived solid tumors

**FIGURE 5:**
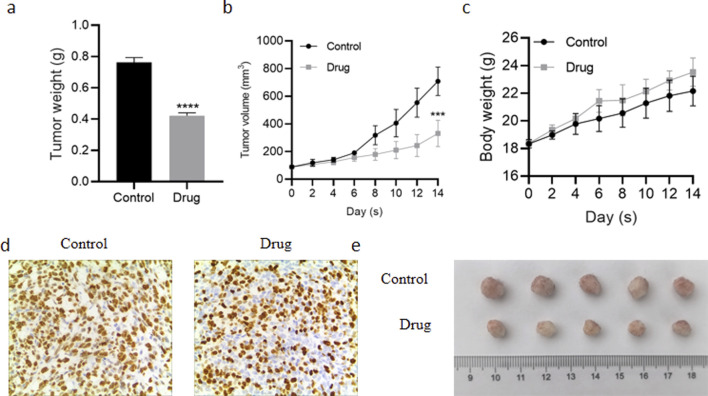
*In vivo* anti-tumor effect of the herbal extract (3:1 ratio) in BALB/c-nude mouse xenograft model. **(a)** Tumor weight at the end of the experiment (n=6). **(b)** Tumor volume measured every other day (n=6). **(c)** Body weight of mice during the treatment period (n=6). **(d)** Representative Ki67 immunohistochemistry images of tumor tissues. **(e)** Photographs of dissected tumors. Data are presented as mean ± SD. Statistical analysis: independent samples t-test for tumor weight and volume comparisons between groups. ***P < 0.001, ****P < 0.0001 compared with the control group.

The *in vitro* experiments have confirmed that Polygonatum sibiricum-Hedyotis diffusa herbal extract at a ratio of 3:1 can inhibit the proliferation of A549 cells. Subsequently, *in vivo* animal experiments were conducted to further verify the effect of this herbal extract on non-small cell lung cancer (NSCLC). As shown in Figures 5a,b, compared with the control group, both the tumor weight and tumor volume of the treatment group were significantly reduced (tumor volume: 708.7 ± 103.4 mm^3^ in the control group vs. 331.3 ± 95.1 mm^3^ in the treatment group, *P* < 0.001; tumor weight: 0.76 ± 0.03 g in the control group vs. 0.42 ± 0.02 g in the treatment group, *P* < 0.0001). The changes in mouse body weight indicated that Polygonatum sibiricum-Hedyotis diffusa herbal extract at a ratio of 3:1 exerted almost no side effects on mice (Figure 5c). In addition, compared with the control group, the Ki-67 positive expression rate was decreased after treatment, suggesting that the herbal extract inhibited the proliferative activity of tumor cells (Figure 5d). The tumor images also intuitively confirmed this result (Figure 5e).

**FIGURE 6:**
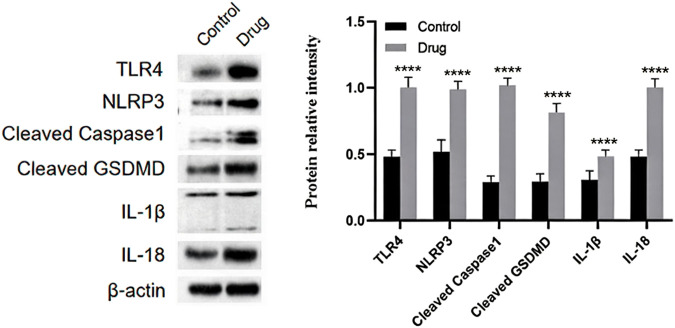
Western blot analysis of TLR4, NLRP3, Caspase-1, GSDMD, IL-1β, and IL-18 protein expression in tumor tissues from the xenograft model (n = 3 mice per group). Data are presented as mean ± SD. Statistical analysis: independent samples t-test. ****P < 0.0001 compared with the control group.

Western blot assay was performed again to evaluate the effect of Polygonatum sibiricum-Hedyotis diffusa herbal extract (3:1) on the expression of pyroptosis-related proteins in tumor tissues. The results in Figure 6 showed that, compared with the control group, the expression levels of TLR4, NLRP3, Caspase-1, GSDMD, IL-1β and IL-18 in the tumor tissues of the treatment group were significantly upregulated, with statistically significant differences (*P* < 0.0001).

## 5 Discussion

As natural medicines, traditional Chinese medicines (TCMs) exert anti-tumor effects through holistic regulation, multi-component and multi-target actions. They can modulate tumor cell proliferation, apoptosis, and the host immune microenvironment, while significantly reducing toxic side effects caused by chemotherapeutic drugs, such as liver and kidney injury and myelosuppression. This meets the core clinical demand of “reducing toxicity and enhancing efficacy” in cancer treatment. Therefore, research on the anti-tumor effects of TCMs has increasingly become a hotspot (Guo et al., 2019; Cheng et al., 2016).

Both Polygonatum sibiricum and Hedyotis diffusa have been proven to exert inhibitory effects on various tumors. Among them, Polygonatum sibiricum mainly functions to strengthen healthy qi, and its polysaccharide components regulate the host immune system. Hedyotis diffusa is more effective in eliminating pathogenic factors, and its terpenoids and flavonoids can directly target tumor cells. However, studies on the mechanism of action and optimal compatibility ratio of their combination in the treatment of non-small cell lung cancer remain scarce.

Pyroptosis is a novel type of programmed cell death accompanied by inflammatory responses. Unlike apoptosis, which is non-inflammatory, pyroptosis activates the host innate immune response by releasing pro-inflammatory factors, achieving the dual effects of eliminating tumor cells and activating anti-tumor immunity (Lin et al., 2015; Huang et al., 2016). Existing studies have confirmed that pyroptosis is closely related to the NLRP3 inflammasome. As a key upstream target in the pyroptosis pathway, TLR4 activates the NLRP3 inflammasome by promoting NF-κB activation, thereby enhancing the release of pro-inflammatory cytokines such as IL-18 and IL-1β. Meanwhile, TLR4 activation mediates Caspase-1-dependent GSDMD activation, ultimately triggering pyroptosis (Zhang and Ma, 2017; Zhu et al., 2024; Yuan et al., 2021; Ye and Hu, 2020). Currently, drugs targeting pyroptosis can overcome the limitations of traditional anti-tumor agents and represent a potential new strategy for cancer treatment.

The positive Annexin V/PI signals and upregulation of pyroptosis pathway markers observed in this study are not contradictory, but reflect a close crosstalk between pyroptosis and apoptosis during tumor cell death induced by the 3:1 Polygonatum sibiricum–Hedyotis diffusa medicated serum. Annexin V/PI staining is a classic method for detecting phosphatidylserine externalization (a common feature of early apoptosis and pyroptotic cells) and loss of cell membrane integrity (a hallmark of late cell death). The 23.77% apoptotic rate detected by flow cytometry in this study represents a comprehensive manifestation of multi-stage cell death phenotypes triggered by pyroptosis activation, rather than pure caspase-dependent apoptosis. Pyroptosis is initiated by activation of the TLR4/NLRP3/Caspase-1 pathway, which cleaves GSDMD to form membrane pores. In the early stage of this process, cells only exhibit phosphatidylserine externalization (Annexin V+/PI−) without complete lysis. In the late stage, enlarged membrane pores lead to PI penetration (Annexin V+/PI+), and this feature is captured as an “apoptotic signal” by Annexin V/PI staining (Zhu et al., 2024; Ye and Hu, 2020). Meanwhile, pro-inflammatory factors such as IL-1β and IL-18 released by pyroptotic cells can further induce secondary apoptotic signals in surrounding A549 cells, also increasing the Annexin V/PI positive rate. The significantly upregulated protein expression of TLR4, NLRP3, Caspase-1, and GSDMD detected by Western blot at both cellular and tissue levels represents the core molecular mechanism of tumor cell death induced by the medicated serum, while the Annexin V/PI signal is a phenotypic reflection of pyroptosis-mediated cell death. This phenomenon is consistent with reports in the literature indicating extensive crosstalk between pyroptosis and apoptosis, and that pyroptosis activation is often accompanied by apoptosis-like phenotypic changes in tumor cells (Lin et al., 2015; Huang et al., 2016).

The combined use of Polygonatum sibiricum and Hedyotis diffusa shows significant synergistic advantages over single-drug administration. Combined with analysis of relevant references, the results of this study fill several gaps in TCM research against non-small cell lung cancer. Liu et al. (2024) reported that Polygonatum sibiricum combined with Scutellaria baicalensis could induce apoptosis in A549 cells by downregulating PON3-mediated mitochondrial damage and endoplasmic reticulum stress. Wang et al. (2024) found that Polygonatum sibiricum combined with Astragalus membranaceus inhibited lung cancer progression through the apelin-PGC1α-UCP1 pathway. Both studies focused on the regulation of apoptosis or metabolic signals by Polygonatum sibiricum combined with other TCMs, without involving the pyroptosis pathway or exploring the optimal compatibility ratio—a key factor affecting clinical efficacy in TCM compound prescriptions. In addition, Zhu et al., 2024, Yuan et al. (2021) confirmed that the monomeric compounds reniformin A and cucurbitacin B could inhibit non-small cell lung cancer progression by inducing TLR4/NLRP3/Caspase-1/GSDMD-dependent pyroptosis, verifying the anti-tumor potential of this pyroptosis pathway. However, such studies only focused on isolated chemical molecules and ignored the synergistic anti-tumor effects of TCM compound prescriptions based on the principle of “strengthening healthy qi and eliminating pathogenic factors”. This study is the first to report that the combination of Polygonatum sibiricum (a classic qi-tonifying herb) and Hedyotis diffusa (a classic heat-clearing and detoxifying herb) can activate the same pyroptosis pathway, and the optimal compatibility ratio of 3:1 was screened through gradient ratio experiments, clarifying the most effective drug ratio for this combination to exert anti-non-small cell lung cancer effects. Different from the above studies, this study not only verified the inhibitory effects of the medicated serum on the proliferation, migration, and invasion of A549 cells *in vitro*, but also confirmed its *in vivo* anti-tumor activity and biological safety in a nude mouse xenograft model (no abnormal weight loss or organ damage in mice). It further revealed the synergistic regulatory mechanism of the two herbs on the pyroptosis pathway at the molecular level: they do not simply produce additive effects of single drugs, but jointly upregulate the TLR4/NLRP3 axis to trigger pyroptosis, which is a concrete manifestation of TCM compatibility principles in modern molecular pharmacology.

This study confirmed that the 3:1 Polygonatum sibiricum–Hedyotis diffusa medicated serum significantly inhibits the proliferation, migration, and invasion of A549 cells, and effectively induces pyroptosis-mediated cell death. Meanwhile, *in vivo* mouse tumorigenesis experiments further verified that this medicated serum markedly delays the growth of non-small cell lung cancer solid tumors. No abnormal body weight loss or organ damage was observed in mice during the experiment, suggesting both anti-tumor activity and biological safety. Furthermore, this medicated serum upregulated the protein expression levels of TLR4, NLRP3, Caspase-1, GSDMD, IL-1β, and IL-18 at both cellular and tissue levels, clarifying its targets in regulating the pyroptosis pathway.

This study only selected A549 lung adenocarcinoma cells as the research model, mainly to ensure the depth and consistency of the mechanistic study on pyroptosis and avoid interference from heterogeneity among different cell lines. In addition, the *in vivo* tumorigenesis experiment further verified the anti-tumor effect at the organismal level. Although this study clarified the mechanism by which the 3:1 Polygonatum sibiricum–Hedyotis diffusa combination exerts anti-non-small cell lung cancer effects through the TLR4/NLRP3/Caspase-1/GSDMD pyroptosis pathway, only a single cell line (A549 lung adenocarcinoma cells) was used for *in vitro* and *in vivo* experiments, without covering other pathological subtypes of non-small cell lung cancer such as lung squamous cell carcinoma and large cell carcinoma. Tumor cells of different subtypes exhibit significant heterogeneity in molecular characteristics, signaling pathway activation patterns, and drug sensitivity. Therefore, the universality of the conclusions of this study needs to be further verified in more non-small cell lung cancer cell lines and corresponding animal models.

Limitations regarding pathway validation:It should be emphasized that all molecular evidence presented in this study is based on protein expression correlations. We did not perform any pathway intervention experiments, such as using specific inhibitors (e.g., TAK-242 for TLR4, MCC950 for NLRP3, VX-765 for Caspase-1) or genetic knockdown approaches (e.g., GSDMD siRNA/shRNA). Therefore, we cannot conclude that the TLR4/NLRP3/Caspase-1/GSDMD axis is necessary for the pyroptosis induced by the Polygonatum sibiricum-Hedyotis diffusa combination. Our results should be interpreted as follows: this pathway is significantly activated after treatment and is associated with the observed pyroptotic phenotype. Definitive causal evidence requires future intervention studies when conditions permit. This is the major limitation of the present study.

Limitations regarding cytokine secretion detection:It should be noted that this study did not measure the secreted levels of IL-1β and IL-18 in cell culture supernatants or animal plasma. Although we detected upregulation of mature IL-1β and IL-18 proteins in cell lysates, which supports inflammasome and caspase-1 activation, direct evidence of cytokine release—a hallmark of pyroptosis—has not been provided. Therefore, we cannot definitively conclude that the observed cell death is canonical pyroptosis. Strictly speaking, our data indicate “pyroptosis-related molecular eventsˮ rather than functionally confirmed pyroptosis. Future studies should verify IL-1β and IL-18 secretion by ELISA or cytometric bead array, and lactate dehydrogenase (LDH) release assays could further confirm membrane pore formation.

It should be noted that multiple comparisons in Figure 1c (various ratio groups vs. control) were not corrected for multiplicity. However, the inhibitory effect of the 3:1 ratio group was extremely significant (P < 0.0001), and even under the most stringent Bonferroni correction (corrected threshold P < 0.0056), the difference remained highly significant. Therefore, the lack of correction does not affect the conclusion that the 3:1 ratio is the optimal combination.

## 6 Conclusion

This study found that the combination of Polygonatum sibiricum and Hedyotis diffusa can enhance the anti-proliferative effect on A549 cells, and determined that the optimal ratio of the two herbs is 3:1. Both *in vitro* and *in vivo* experiments have demonstrated that Polygonatum sibiricum-Hedyotis diffusa herbal extract at a ratio of 3:1 exerts inhibitory effects on NSCLC A549 cells and solid tumors accompanied by pyroptosis and upregulation of TLR4, NLRP3, Caspase-1, GSDMD, IL-1β, and IL-18 proteins. These results suggest that the TLR4/NLRP3/Caspase-1/GSDMD pathway may be involved in the observed anti-tumor effects, but causal validation (e.g., using specific inhibitors or gene knockdown) is required in future studies. This study provides a new idea for the treatment of non-small cell lung cancer.

## Data Availability

The original contributions presented in the study are included in the article/supplementary material, further inquiries can be directed to the corresponding author.
